# Predicting Frequency from the External Chemical Environment: OH Vibrations on Hydrated and Hydroxylated Surfaces

**DOI:** 10.1021/acs.jctc.2c00135

**Published:** 2022-12-02

**Authors:** Andreas Röckert, Jolla Kullgren, Kersti Hermansson

**Affiliations:** Department of Chemistry, Ångström Laboratory, Uppsala University, Uppsala751 21, Sweden

## Abstract

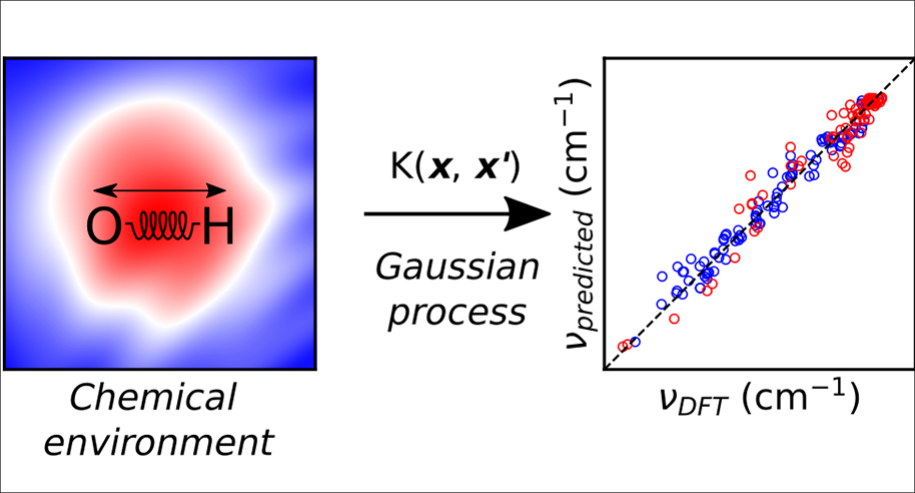

Robust correlation curves are essential to decipher structural information from IR-vibrational spectra. However, for surface-adsorbed water and hydroxides, few such correlations have been presented in the literature. In this paper, OH vibrational frequencies are correlated against 12 structural descriptors representing the quantum mechanical or geometrical environment, focusing on those external to the vibrating molecule. A nonbiased fitting procedure based on Gaussian process regression (GPR) was used alongside simple analytical functional forms. The training data consist of 217 structurally unique OH groups from 38 water/metal oxide interface systems for MgO, CaO and CeO_2_, all optimized at the DFT level, and the fully anharmonic and uncoupled OH vibrational signatures were calculated. Among our results, we find the following: (i) The intermolecular *R*(H···O) hydrogen bond distance is particularly strong, indicating the primary cause of the frequency shift. (ii) Similarly, the electric field along the H-bond vector is also a good descriptor. (iii) Highly detailed machine learning descriptors (ACSF, SOAP) are less intuitive but were found to be more capable descriptors. (iv) Combinations of geometric and QM descriptors give the best predictions, supplying complementary information.

## 1 Introduction

Structure–property correlations are valuable guides toward identification of structural candidates (motifs) hidden in experimental signatures, and the exclusion of unlikely ones. For example, vibrational spectroscopy measurements combined with frequency–structure correlations constitute one of the preferred approaches to help locate protons and their structural motifs in solids where diffraction experiments are unfeasible. One such case is the determination of minor amounts of H atoms in “nominally anhydrous minerals”,^1^ which play a major role in the water circulation on, in, and above Earth. We will give references to many published correlation studies in this paper; they are placed in their proper contexts within section 3, Results and Discussion.

Our paper deals specifically with water on metal oxide surfaces. Be it in the presence of a thin or a thick water film, the water/solid interface region is particularly challenging to explore by experiment due to the relatively few water molecules involved, the multitude of plausible local adsorption modes, and the particular structural features arising from the 2D nature of the system and the solid’s templating effect. Additionally, the insulating or semiconducting character of the oxide and any special selection rules for the systems and techniques in use need to be considered.

Vibrational spectroscopy techniques for interfaces are fraught with the difficulties listed above, but even so, these methods are among the most sensitive available to gain information about the structure of water–metal oxide interfaces.^2,62^ Here we will use theoretical calculations to investigate which structural features (descriptors) external to the interfacial OH species that are best mirrored in the vibrational frequency signatures. Or, put in another way, given a geometric or electronic structure surrounding the OH oscillator, *how well can we predict the vibrational frequency?*

**Figure 1:**
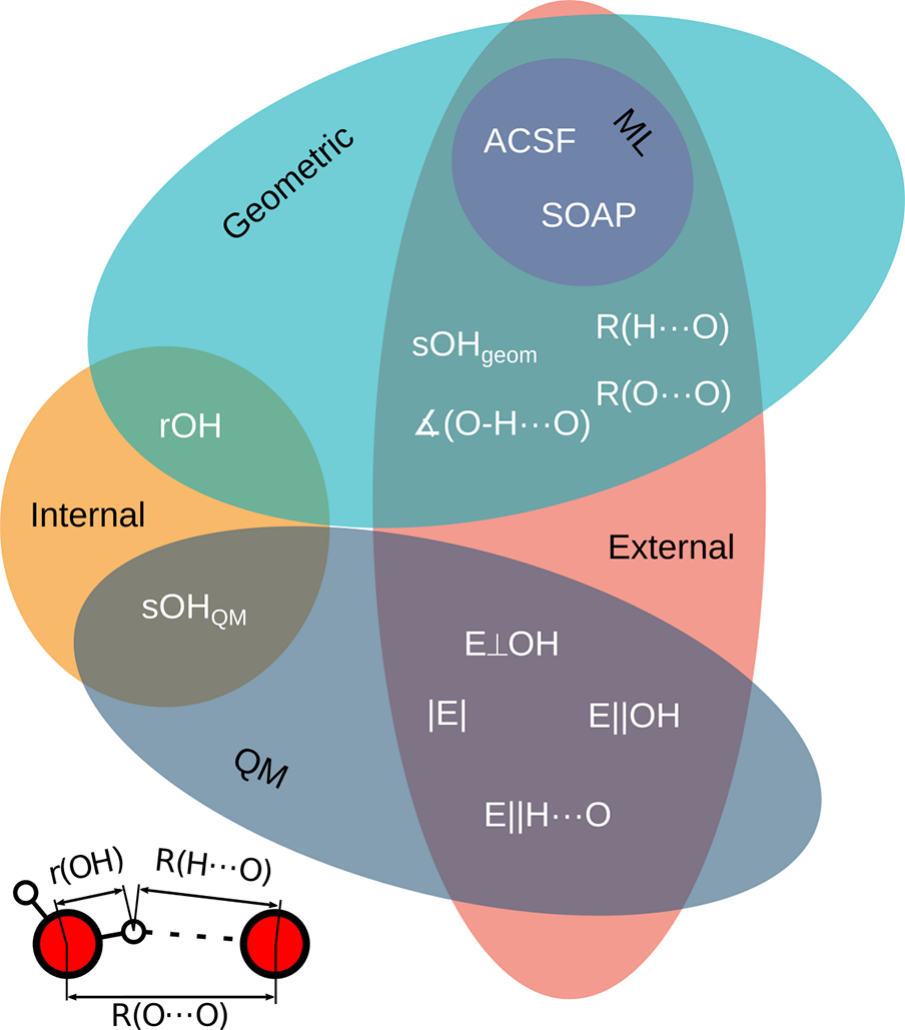
Overview of descriptor classes relevant for OH oscillators in chemical environments and used in the present study. The external environment surrounding a water molecule or a hydroxide group in the interface region is represented using geometric, quantum mechanical (QM), and machine-learning (ML) descriptors. The internal (intramolecular) OH bond distance, *r*(OH), and the intramolecular QM-determined bond-order, *s*(OH)_QM_, are special in that they are representations of the OH oscillators themselves but not their surroundings. See text for more details. The small image in the bottom left corner shows a schematic image of a typical hydrogen bond with key distances marked.

**Figure 2:**
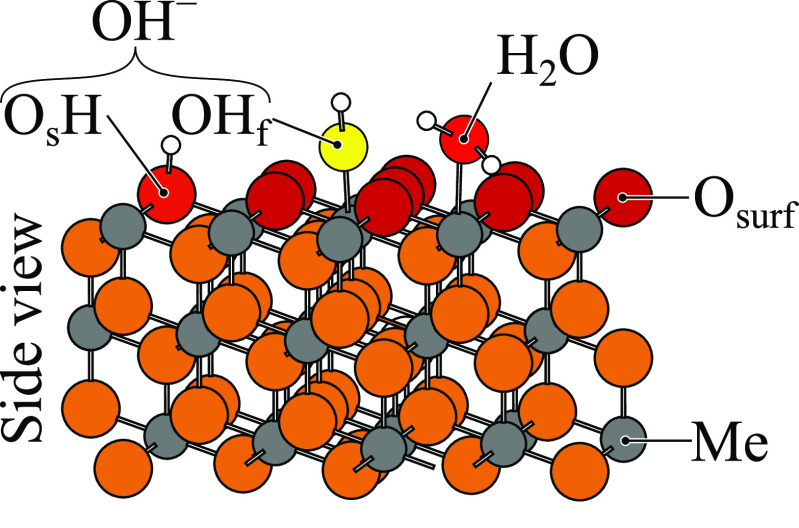
Surface water species typically found on hydrated/hydroxylated metal oxide surfaces: intact H_2_O molecule, and dissociated water, which gives rise to hydroxide ions of the O_s_H or OH_f_ kinds (the negative charge is seldom written out in this notation). The s in O_s_ stands for “surface”, and the f in H_f_ stands for “free”. Some of the water OH groups are non-H-bonded or “dangling”. Typical hydrogen-bonded formations on the surface are HOH···OH_2_, HOH···O^2–^, HOH···OH_f_, and O_s_H···OH_f_.

Overall, considerable effort has been made in the scientific community to construct vibrational frequency−structure correlation curves for water and OH^–^ groups; see references in Section 3.2. As far as we are aware, all such experimental correlations (and almost all theoretical ones) focus on bulk systems. Given that the majority of surfaces (of all categories) are hydrated/hydroxylated under ambient conditions, access to such relations for surface water will fill a knowledge gap.

All frequencies presented in this paper refer to stretching OH-oscillators of intact or dissociated surface water, generated using density functional theory (DFT) calculation. The vibrational signatures are sampled at many different water coverages for hydrated and hydroxylated overlayers on CeO_2_ (ceria) (111), MgO(001), and CaO(001), three oxides of rather different chemical nature. All structures were fully optimized. Some data were newly generated for this study; others were taken from earlier studies (see Method section).

Figure 1 lists the structural descriptors that we will use for all the surface OH-oscillators in this paper. The descriptors can be divided into internal and external descriptors. We consider each OH oscillator as surrounded by a perturbing external environment and focus on the external descriptors, that is, as said above, the descriptors of the surroundings of each water molecule (for intact water molecules) or of each hydroxide ion (for dissociated water molecules); see the schematic illustration of a water/metal oxide interface system in Figure 2. Among the external descriptors, we first present results for single physics-based descriptors, starting with the geometrical H-bond distances commonly used for condensed matter, and then bond orders and electric field descriptors determined from the electron density, thereby the category quantum-mechanical (QM) descriptors in Figure 1. Next all combinations of two physical descriptors are presented, and finally the machine-learning (ML) atom-based descriptors, ACSF (atom-centered symmetry functions)^3^ and SOAP (smooth overlap of atomic positions),^4,5^ which also operate on the geometry of the system. These two are frequently used in different schools of interatomic potential generation through machine learning (ML); see refs (6 and 7).

The organization of the paper is as follows. Systems and methods are discussed in section 2, conclusions are given in section 4, and between these is section 3, Results and Discussion, arranged according to the following:

section 3.1, Scatter plots for all the descriptors
section 3.2, Single descriptors
section 3.2.1, Single geometrical descriptors
section 3.2.2, Bond-order descriptors
section 3.2.3, Electric field descriptors
section 3.3, Two descriptors
section 3.4, ML descriptors: SOAP and ACSF
section 3.5, internal *r*(OH) distance, a special case

The ability of each descriptor to predict the vibrational frequency is evaluated using an approach based on Gaussian process regression (GPR), which enables fitting without a prerequisite function describing the correlation. In addition to fitting to vibrational data by means of GPR, simple analytical expressions are also least-squares-fitted for the single, physics-based descriptors, providing additional insight and simplicity.

The discussion of the intramolecular OH bond length as an internal descriptor is postponed until the end of section 3 as, akin to the OH vibrational frequency, it is rather a probe of the surroundings than a descriptor of it.

## 2 Methods

### 2.1 Workflow, Data Sets, and Systems

**Figure 3:**
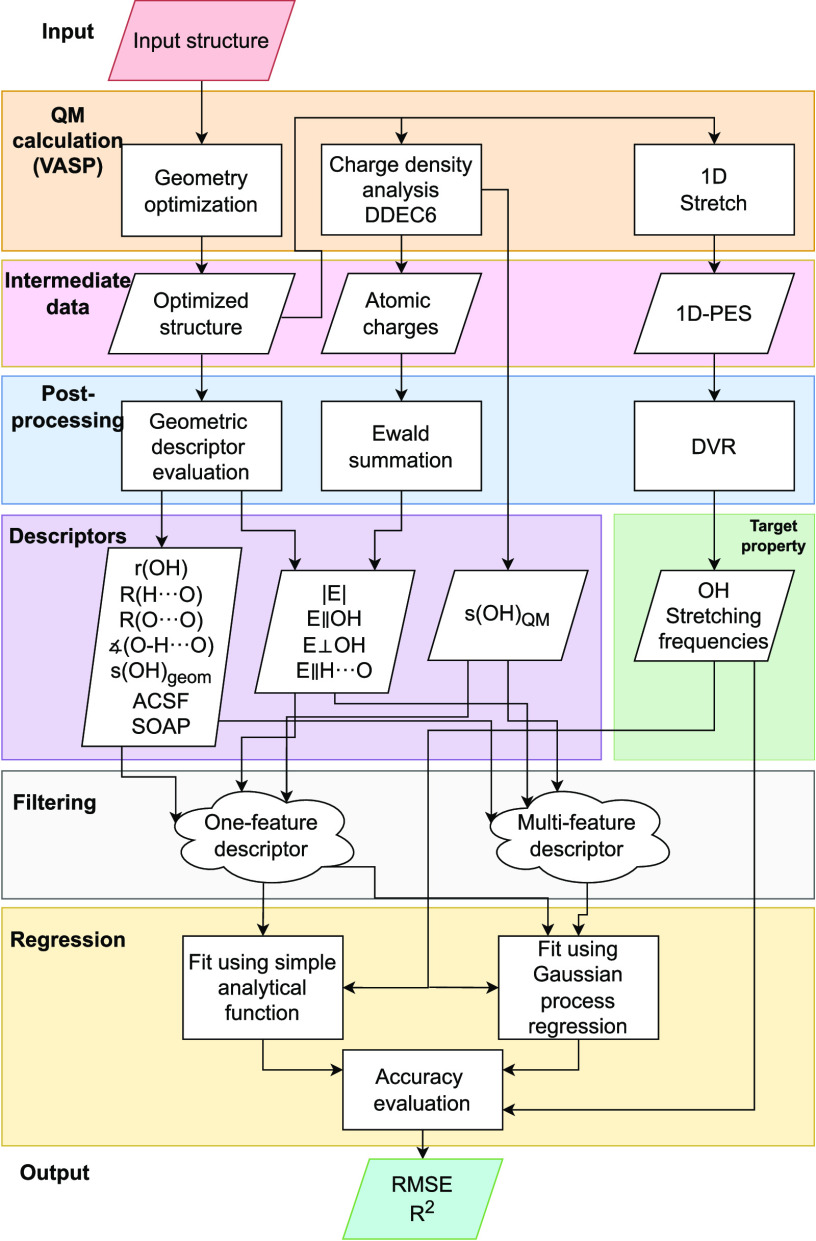
Our workflow as described in section 2.

The overall workflow, involving data generation and postprocessing data analytics with respect to the selected descriptors, is shown in Figure 3 and further elaborated in sections 2.2–2.5. All data presented in the figures and tables have been calculated with the same DFT functional: optPBE-vdW. The systems included in this work are listed in Table 1. Altogether, results from 217 structurally unique OH groups are analyzed. The chemical environments of our H_2_O and OH^–^ species exhibit a broad range of hydrogen bond environments and strengths, which is reflected in the wide range of vibrational frequencies covering the range 3600 cm^–1^ to 2000 cm^–1^ in the calculations. Both intact and dissociated water molecules occur on all three oxide surfaces.

**Table 1:** List of the Water/Metal Oxide Systems Included in the Current Study

surface	water coverages	total no. of structurally unique water molecules (intact or dissociated)	no. of unique OH groups investigated		
			O_w_H	O_s_H	OH_f_
CeO_2_(111)	1-,2-mers,1 ML (many),1.5 ML	65	68	29	29
MgO(001)	1-, 2-, 3-mers,1 ML, 1.25 ML	12	16	4	4
CaO(001)	1-, 2-, 3-mers,1 ML, 1.25 ML	34	41	13	13

For ceria, the majority of the structures were taken from the optPBE-vdW-optimized ceria surface structures generated in ref (9); additionally a number of new structures were generated in the present work. The MgO(100) and CaO(100) structures were all taken from ref (8). Below we only briefly summarize the procedures of the structure generation and refer to the cited publications for more details.

#### 2.1.1 Water/Ceria(111) Systems

The slab systems are composed of four O–Ce–O triple layers, that is, 12 atomic layer thick slabs separated by 30 Å of vacuum, perpendicular to the surface. To allow for different translational symmetries of the H-bond patterns of the H_2_O/OH^–^ overlayer, five supercells were used, corresponding to p(1 × 1), p(1 × 2), p(1 × 4), p(2 × 2), and p(3 × 3) expansions of the primitive surface unit cell.

Water was adsorbed on one of the two surfaces at coverages ranging from isolated monomers to 1.5 ML. All systems were charge neutral and contained the following OH-species: H_2_O, OH_f_, and O_s_H (see definitions in Figure 2). For many coverages, multiple (meta)stable structures are present in our data. For more information, see ref (9).

#### 2.1.2 Water/MgO(001) and Water/CaO(001) Systems

Here, the slab systems are composed of 4 atomic layer thick slabs separated by 15 Å of vacuum, perpendicular to the surface. Three different supercells were used to allow for different translational symmetries of the H-bond patterns of the H_2_O/OH^–^ overlayer, as listed in Table 1 of ref (8). Water was adsorbed on one side of the slab in coverages ranging from isolated water molecules to 1.25 ML coverage. For CaO(001), all coverages contain dissociated water molecules (with or without intact water molecules); for MgO(001), dissociation occurs only from the 3-mer and onward.

#### 2.1.3 Previous Theoretical Work

A considerable number of theoretical studies have been performed for water on ceria(111) (see the comprehensive list of references in refs (9−16)), as well as for MgO(001) (see the list in ref (8) and some very recent ones in refs (17−23)) and for CaO (see list of references in ref (8)). As for the descriptors, we note that four of the descriptors (*R*(O···O), *R*(H···O), the angle ∠(O–H···O), and *E*∥OH) discussed in detail in this paper were also discussed for MgO(001) and CaO(001) in refs (8 and 24). respectively.

### 2.2 Electronic Structure Calculations

The calculations were performed using the Vienna *Ab initio* Simulation Package^25−28^ (VASP 5.4.4) using the optPBE-vdW functional.^29,30^ The valence electrons Ce(4f,5s,5p,5d,6s), Mg(2p,3s), Ca(3p,4s), O(2s,2p), and H(1s) were treated explicitly, and the core electrons were treated with the projector-augmented wave (PAW) method, and spin polarization was not invoked. The plane-wave bases were truncated at 400, 520, and 600 eV for crystals containing Mg, Ce, and Ca, respectively.^a^ The criterion in the self-consistent-field cycle was set to 10^–7^ and 10^–6^ eV for CeO_2_ and MgO/CaO, respectively. The Brillouin zone was sampled with a grid of points with a maximum spacing between k-points along any reciprocal cell axis of 0.086 Å^–1^. Gaussian smearing of 0.1 eV was applied.

### 2.3 Vibrational Analysis

OH stretching vibrational frequencies were calculated using a 1D uncoupled OH vibrational model with the bond-stretching protocol described in ref (31). The main advantage of using a 1D vibrational model is the same as that used by experimental practitioners in the isotope-isolation technique: to create uncoupled modes that highlight the effects of the local environment on one particular molecular bond at a time. While controlled experiments for isotope-isolated systems require some additional preparation compared to fully deuterated or protonated samples, the uncoupled bond stretching protocol is relatively straightforward to carry out using modeling.

Our method is as follows. From the optimized geometry of the system, one OH group was stretched and contracted around its center of mass at 71 equidistantly placed points in the range [−0.375, +0.675] Å from the equilibrium position. Simultaneously, the rest of the system remained fixed and the 1D potential energy surface (PES) of the flexible oscillator was generated. The masses of O and H were set to 15.994915 amu and 1.007825 amu, respectively. The 1D vibrational Schrödinger equation was then solved for the vibrational eigenvalues using the discrete variable basis-set representation (DVR) following Light et al.^32,33^ The fundamental vibrational wavenumber (ν) was calculated from the energy difference between the ground and the first excited vibrational state (see further ref (31)).

We report said vibrational energy difference without applying any scaling or correction factors. For both the water and the hydroxide species, our DFT-calculated gas-phase values lie approximately 100 cm^–1^ below the experimental values, which is consistent with other GGA functionals that we have tested.^8,9,34^ More precisely, our calculated anharmonic frequency gas-phase value is 3578 cm^–1^ for OH in HDO water and 3459 cm^–1^ for the hydroxide ion, which means that they are downshifted by 129 and 97 cm^–1^ compared to the experimental frequencies of refs (35 and 36), respectively.

### 2.4 ML Descriptors

Twelve descriptors of the chemical environment are used in the paper. These are divided into internal, geometric, quantum (QM), and machine learning (ML) descriptors (Figure 1). They are all described in their proper places in section 3, except for SOAP^4,5^ and ACSF,^3^ which are treated in this section as they require more elaboration of the technicalities related to the selection of parameters and settings that we have made. Somewhat casually, we refer to them as ML descriptors as they were primarily designed by their developers to be used to generate machine-learned potentials.

We have used the Dscribe package^37^ to calculate features representing the local environment. In these calculations, the hydrogen position of the targeted OH oscillator served as our origin, and we have *excluded the whole OH oscillator from the environment when surface OH^–^ species are targeted and the whole water molecule when the OH oscillator belongs to a water molecule.*

The smooth overlap of atomic positions expresses the local atomic environment around an atom in terms of neighbor densities defined as a superposition of Gaussian functions with a predefined standard deviation, σ (in the current work, we used a value of 1 Å), centered on each of the neighboring atoms. These densities, in turn, are approximated by an expansion of radial basis functions (RBFs) and spherical harmonics. The number of radial basis functions (*n*_r_) and the maximum degree of spherical harmonics (*n*_l_) define the quality of the representation by controlling the number of elements in the so-called partial power spectrum vector,^4^*p̅*, which constitutes the feature vector used in the SOAP formalism. We used the default settings of Dscribe with RBFs defined by spherical Gaussian type orbitals as described in ref (5) with *n*_l_ and *n*_r_ values in the ranges of (0–4) and (1–4), respectively.

ACSF can also characterize the neighbor densities in a local environment around a central atom, i.e. in our case, the hydrogen of the OH oscillator. The ACSF used in the current work corresponds to the so-called G1 and G2 functions in the original Behler–Parrinello approach.^3^ These Gaussian functions describe neighbor densities in spherical shells at different radii surrounding a central atom. The set of Gaussian functions constitutes the feature vector, each tuned to different characteristic distances. These shells are normally (and also in this work) equidistantly placed between the atomic center and the cutoff radius, *r*. The standard deviation of each of the *n* Gaussian functions was here set to  corresponding to a full width at half-maximum of . We used values of *n* in the range of 2–9.

Here we use two ML descriptors: ACSF and SOAP. A comprehensive list of descriptors used in atomistic machine learning were recently reviewed in ref (38).

### 2.5 The Regression Procedures

In order to treat the various descriptors on an equal footing, we used a Gaussian process regression (GPR) to fit correlation functions, that is, ν(OH) vs descriptor relations. For each descriptor, we have performed two separate regressions: one for the water data-set and one for the hydroxide-data-set. We have used the Scikit-learn package^39^ (version 1.0.2) to perform the regression.

We used the same kernel, *K*(**x**_*i*_, **x**_*j*_), in all cases, namely, the dot-product kernel with an exponentiation of 4:
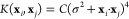
1

Tikhonov regularization was used with an α value of (50)^2^ (cm^–1^)^2^. This choice of α value was based on the observation that for many of the single physical descriptors used here, such an α value was found to minimize the test set’s root-mean-square error and supress overfitting (see Supporting Information for a number of representative examples).

Throughout the paper, we report the quality of our descriptors in terms of the average root-mean-square error (RMSE) and goodness-of-fit value (*R*^2^). These values were calculated from a set of 8 different 80/20 splits for training vs testing over the whole data set. Splitting into only a training and testing set can lead to overfitting, but no such overfitting was established, see Supporting Information. The populations in the test and training sets were selected randomly but with the constraint that the relative occurrences of the three systems, CeO_2_, MgO, and CaO, were equally represented in the test and training sets. The RMSE and *R*^2^ values presented in Section 3 refer to calculations over the test set only. For convenience, we will refer to them as RMSE and *R*^2^ without using any special notation to indicate that we average over 8 sets. In addition, we also calculated the RMSE and *R*^2^ over the populations of OH^–^ and H_2_O species separately. Unless specifically said otherwise, the RMSE and *R*^2^ reported refer to the test data set containing both the OH^–^ and H_2_O data.

## 3 Results and Discussion

The organization of this section was listed at the end of section 1. The main focus lies on the external descriptors (sections 3.1–3.4), and we devote section 3.5 to the internal descriptor *r*(OH).

### 3.1 Scatter Plots for the 12 Descriptors

**Figure 4:**
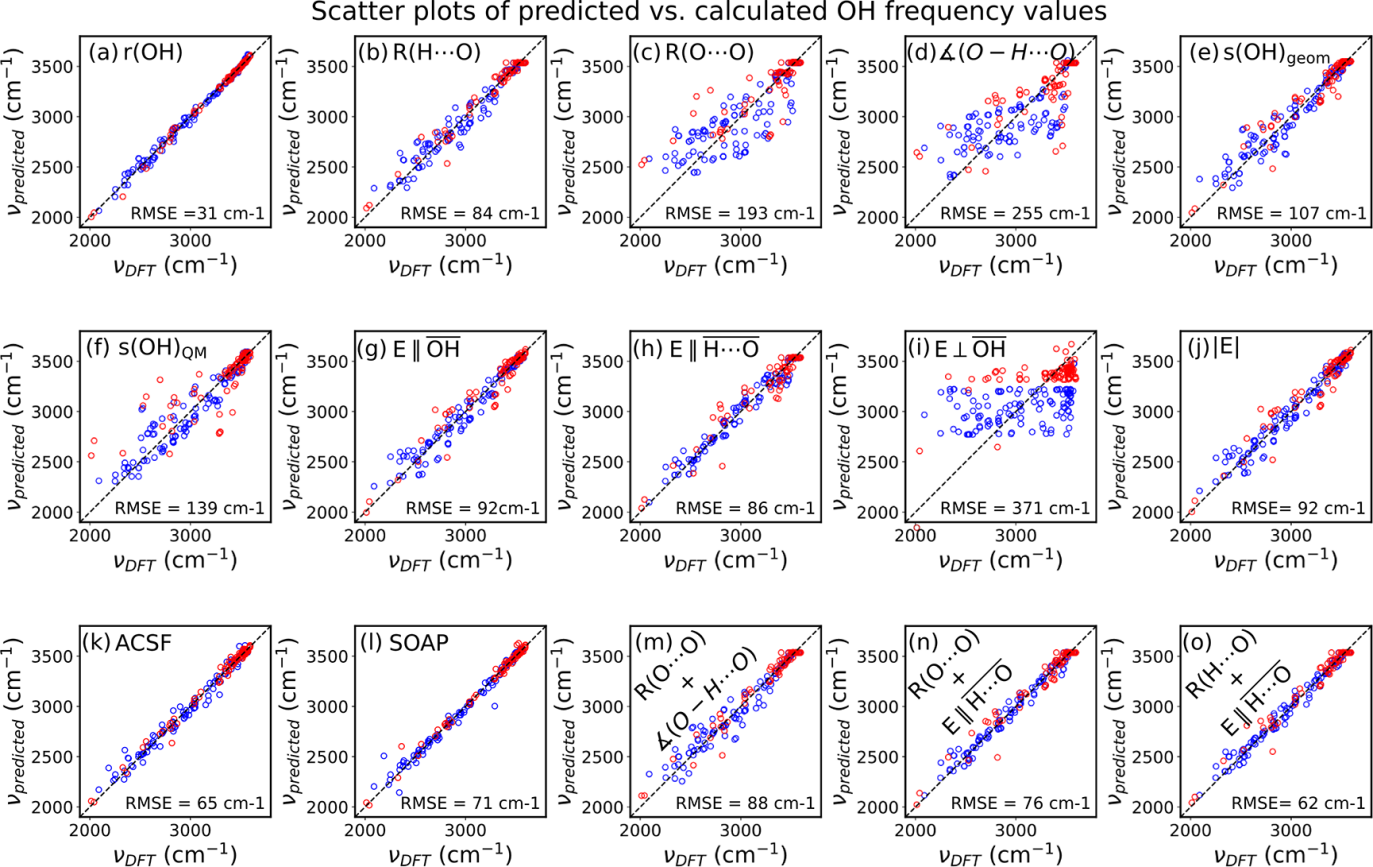
Scatter plots of predicted vs calculated OH frequency values from Gaussian process regression using one or two descriptors in the fitting process. Here and in the following, the data are all from optPBE-vdW DFT calculations for the intact water molecules (blue) and *O*_s_H and O*H*_f_ hydroxides (both red) on CeO_2_(111), MgO(100) and CaO(100). The RMSE deviations are listed in the plots. Separate regressions were performed for the water data-set and the hydroxide data-set. The RMSE value reported in each frame in the figure was calculated over the combined data-set.

The scatter plots in Figure 4 give an overview of how well the descriptors listed in Figure 1 manage to reproduce the DFT-calculated vibrational frequencies in unseen data (test set), given our GPR procedure. The quality of fit using the root-mean-square-error, RMSE, is listed within each frame; the blue marks in the plots refer to OH oscillators in intact water molecules and the red marks to hydroxide ions.

We will discuss each descriptor class in detail in the following, but the scatter plots already convey plenty of information. It is, for example, clear from the figure that *R*(O···O), ∠(O–H···O), and *E*⊥OH are particularly bad at predicting the vibrational frequencies of the surface OH oscillators, while several of the other single descriptors perform very well.

### 3.2 Single Descriptors

#### 3.2.1 External Geometrical H-Bond Descriptors

##### 3.2.1.1 Context

With external geometric H-bond descriptors, we refer to the distances and angle involved in a single hydrogen bond where our oscillator acts as a donor and the acceptor is an oxygen atom (either as part of a water or hydroxide species or in the form of an O^2–^ ion). We denote the distance between the O atoms in the donor and acceptor *R*(O···O) and the distance between the donating hydrogen and the accepting oxygen we denote *R*(H···O). The corresponding O–H–O angle is denoted ∠(O–H···O). Many experimental ν(OH) vs *R*(O···O) or *R*(H···O) correlation curves have been published and are being widely used in the scientific community for analysis of local environments of OH-oscillators in bulk crystals. Some of the more comprehensive correlation plots are those published by Novak (1974),^40^ Berglund et al. (1978),^41^ Bertolasi et al. (1996),^42^ Libowitzky (1999),^43^ and Steiner (2002).^44^ They all refer to crystalline bulk systems where the distances were obtained using X-ray or neutron diffraction. As far as we are aware, no such correlations exist from experimental surface structures, where diffraction techniques are less well resolved. Even for methods that can resolve the atomic positions on the surface, such as atomic force microscopy and scanning tunneling microscopy, these distances are not sufficiently resolved to discuss correlations without modeling efforts. Therefore, it has not yet been demonstrated whether the correlations suitable for bulk systems apply to surface systems. This is one more example where modeling can contribute, and it will be explored here.

Note: In this work, we use the following hydrogen-bond definition. A hydrogen bond is said to exist if *R*(H···O) < 2.5 Å and ∠(O–H···O) > 120°.

##### 3.2.1.2 Results

**Figure 5:**
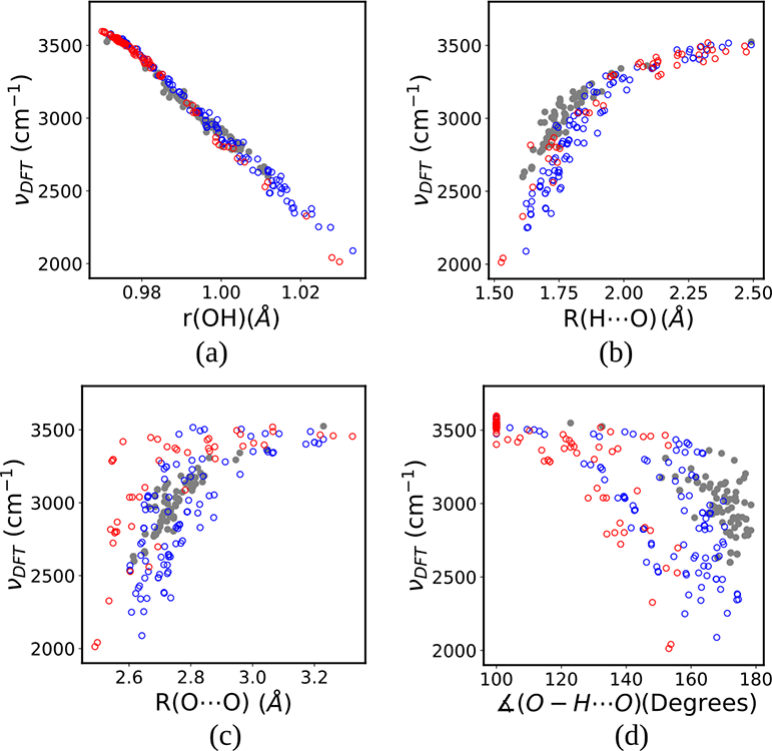
Comparison between surface and bulk OH oscillators in terms of ν(OH) with respect to (a) *r*(OH), (b) *R*(H···O), (c) *R*(O···O), and (d) ∠O–H···O H-bond angle. Surface water (blue rings), surface hydroxides (red rings) and water bound in bulk crystalline hydrates and hydroxides (gray circles) are shown. Bulk data from ref (8).

Starting with the correlation between ν(OH) and *R*(O···O), it is seen to be poor for our surface data (*R*^2^ = 0.696) (see the colored rings in Figure 5c) and much worse than for the experimental bulk systems of Libowitzky,^43^ who reported an *R*^2^ value of 0.96 for his fitted exponential curve. The reason for the mediocre performance of the *R*(O···O) descriptor for the hydrated-hydroxylated surfaces compared to bulk data (gray circles in Figure 5c) is likely due to the many strained geometries with highly bent H-bonds encountered in the surface data compared to bulk data (see Figure 5d). This is also reflected by the fact that, while the angles themselves perform poorly with *R*^2^ = 0.459 and RMSE = 255 cm^–1^ (Figure 4d), a combination of the *R*(O···O) distance and the ∠(O–H···O) angle performs well, with *R*^2^ = 0.950 and RMSE = 88 cm^–1^ (Figure 4m), albeit slightly worse than the single *R*(H···O) descriptor with *R*^2^ = 0.954 and RMSE = 84 cm^–1^ (Figures 4b). In fact, the single *R*(H···O) descriptor performs very well, both for bulk and surfaces (see Figure 5b).^b^

**Figure 6:**
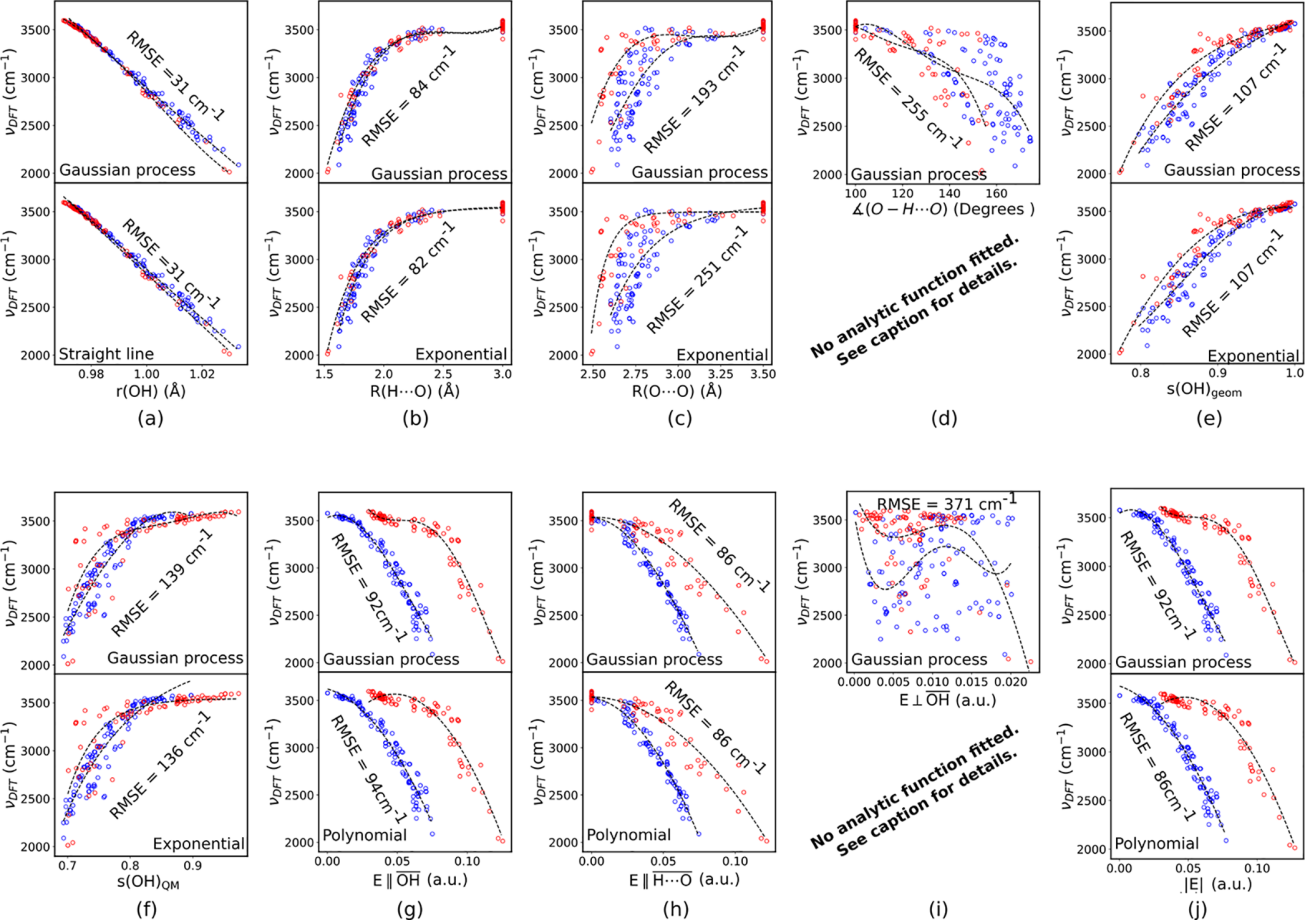
(a–j) Correlations between the DFT-generated frequency values and a selected (single) descriptor variable. The blue dots refer to OH groups belonging to an intact water molecule; the red dots refer to OH groups of hydroxide ions. For each descriptor, there are two frames: the top frame refers to fitting by means of Gaussian process regression (GPR) and the lower frame to fitting with a simple analytical function. The fitted model functions are drawn as dashed black lines. With GPR, the model function displayed is one representative from the set of eight regressions performed based on 80/20 training/testing splits; see section 2.5. For the fitting to an analytical function, a limited set of polynomial/exponential functional forms were explored, and the best functional form found was used for both the blue and the red OH data. The fitted analytical functions are listed in Table 2, together with RMSE deviations for both the GPR and analytical fitting approaches. Given the poor correlation between H-bond angle and frequency in panel d, as well as between E⊥OH and the frequency in panel i, we did not deem it meaningful to generate analytical expressions for these.

The fitted correlation curves, corresponding to the scatter plots in Figure 4b–d, are shown in Figure 6b–d, top frame (GPR). In addition, correlation curves using simple analytical functions are shown in the bottom frames. The mathematical expressions for our best-performing analytic functions are given in Table 2.

**Table 2:** Simple Analytical Functions (AF) of ν(OH) vs. Descriptor Correlation Functions Obtained from Least-Squares Fitting^a^

descriptor	figure no.	pop.	fitted function	*R*^2^ AF	RMSE AF (cm^–1^)	RMSE GPR (cm^–1^)
*r*(OH)	6a	H_2_O	ν(OH) = 28382 – 25486*r*(OH)	0.994	31	31
		OH^–^	ν(OH) = 29692 – 26838*r*(OH)			
*R*(H···O)	6b	H_2_O	ν(OH) = 3552 – 563923 exp[−3.7427*R*(H···O)]	0.955	82	84
		OH^–^	ν(OH) = 3543 – 387690 exp[−3.6398*R*(H···O)]			
*R*(O···O)	6c	H_2_O	ν(OH) = 3573 – 303244509 exp[−3.9096*R*(O···O)]	0.316	251	193
		OH^–^	ν(OH) = 3497 – 1119223148546828 exp[−11.0475*R*(O···O)]			
*s*(OH)_geom_	6e	H_2_O	ν(OH) = 4137 – 343880 exp[−6.4849*R*(O···O)]	0.924	107	107
		OH^–^	ν(OH) = 3691 – 20747231 exp[−12.1364*R*(O···O)]			
*s*(OH)_QM_	6f	H_2_O	ν(OH) = 3914 – 1777661 exp[−10.0496*R*(O···O)]	0.8676	136	139
		OH^–^	ν(OH) = 3579 – 33146109 exp[−15.0278*R*(O···O)]			
*E*∥OH	6g	H_2_O	ν(OH) = 3622 – 196774*E*^2^ – 4240.1077*E*	0.941	94	92
		OH^–^	ν(OH) = 2986 – 254521*E*^2^ + 24325.283*E*			
*E*∥H···O®	6h	H_2_O	ν(OH) = 3535 – 230291*E*^2^ – 2491.1702*E*	0.9493	86	86
		OH^–^	ν(OH) = 3538 – 98376*E*^2^ – 434.02758*E*			
total *E*	6j	H_2_O	ν(OH) = 3676 – 199230*E*^2^ – 4511.8717*E*	0.9493	86	92
		OH^–^	ν(OH) = 3025 – 239817*E*^2^ + 22673.08*E*			

a All quantities were obtained from our DFT calculations. The fitted parameters presented in the table are taken from *one* representative fit. RMSE and *R*^2^ values refer to the whole combined data-set and are given in the rightmost columns. Distances are given in Å, electric fields in atomic units.

##### 3.2.1.3 Summary

For our surface OH groups, the ν(OH) vs *R*(H···O) correlation is strong. In contrast, the surface ν(OH) vs *R*(O···O) correlation is much weaker.

#### 3.2.2 Bond Order Descriptors

##### 3.2.2.1 Context

The topic of bond orders has a long history in science, and we quote Manz (2017):^45^ “Bond order is a widely used concept throughout the chemical sciences. Bond order is widely taught in basic and advanced chemistry courses. Bond order is also widely used in scientific research. A search for ‘bond order’ (with quotation marks) in Google Scholar returned 152 000 results.” Five years later (September 2022), this number is 170 000. The bond order relates to the bond strength between atoms and was initially likened to the “Pauling bond strength”, which is the atomic valence of a cation divided by its coordination number,^46^ but the concept has evolved with time to include more quantitative measures of the binding strength.

In brief, bond order descriptors can be classified into those involving electronic orbitals or electron density (we denote them *s*_QM_) and those expressed by geometric parameters (*s*_geom_). Examples of the former are the Natural Bond Order formalism of Weinhold et al.^47^ and descriptors based on topology features of the electron density landscape according to Bader.^48^ Geometrical bond order descriptors typically express the bond order in terms of bond lengths by way of some generalized expression, based on interatomic bond distances,^46,49,50^ often suitably summed over the central atom’s neighbors.

Examples of correlations between the vibrational OH frequency and the geometric bond order in inorganic bulk crystals are found in refs (51 and 52).

##### 3.2.2.2 Results

In the present report, we determine the intramolecular bond order from the sum over intermolecular bond distances *s*(OH)_geom_ = 1 – *∑*_*i*_ exp[*R*_0_ – *R*(H---O)_*i*_/*B*] for all oxygen atoms within a 7 Å sphere from the targeted H, excluding the O internal to the OH oscillator itself. The parameters *R*_0_ = 0.914 Å and *B* = 0.404 Å described in ref (51) were used, which originate from ref (53) and were fitted to experimental crystallographic data of (close to) linear O–H···O bonds. Note that the model only accounts for interactions between hydrogen and oxygens.

We find that, over our data set, *s*(OH)_geom_ varies in the range from 0.75 to 1. The correlation between ν(OH) and *s*(OH)_geom_ (Figure 6e) is poor with a RMSE of 107 cm^–1^ and is inferior to ν(OH) vs *R*(H···O). The large RMSE could possibly be ascribed to the fact that, unlike *R*(H···O), *s*(OH)_geom_ does not *exclude* O atoms that are found at short distances but with angles outside of our hydrogen-bond definition (∠(O–H···O) > 120°).

We also calculated the O–H bond order from the charge density using the Chargemol/DDEC6 program,^45^ which estimates the bond order, defined as the number of shared electron pairs in a bond, from the overlap of spherically averaged electron densities between the atoms using an Atoms in Molecules (AIM) approach.^49^ The resulting *s*(OH)_QM_ is an internal descriptor and is found in a comparable range (0.66 to 0.97) to *s*(OH)_geom_ but gives a larger RMSE of 139 cm^–1^ (Figure 6f).

**Figure 7:**
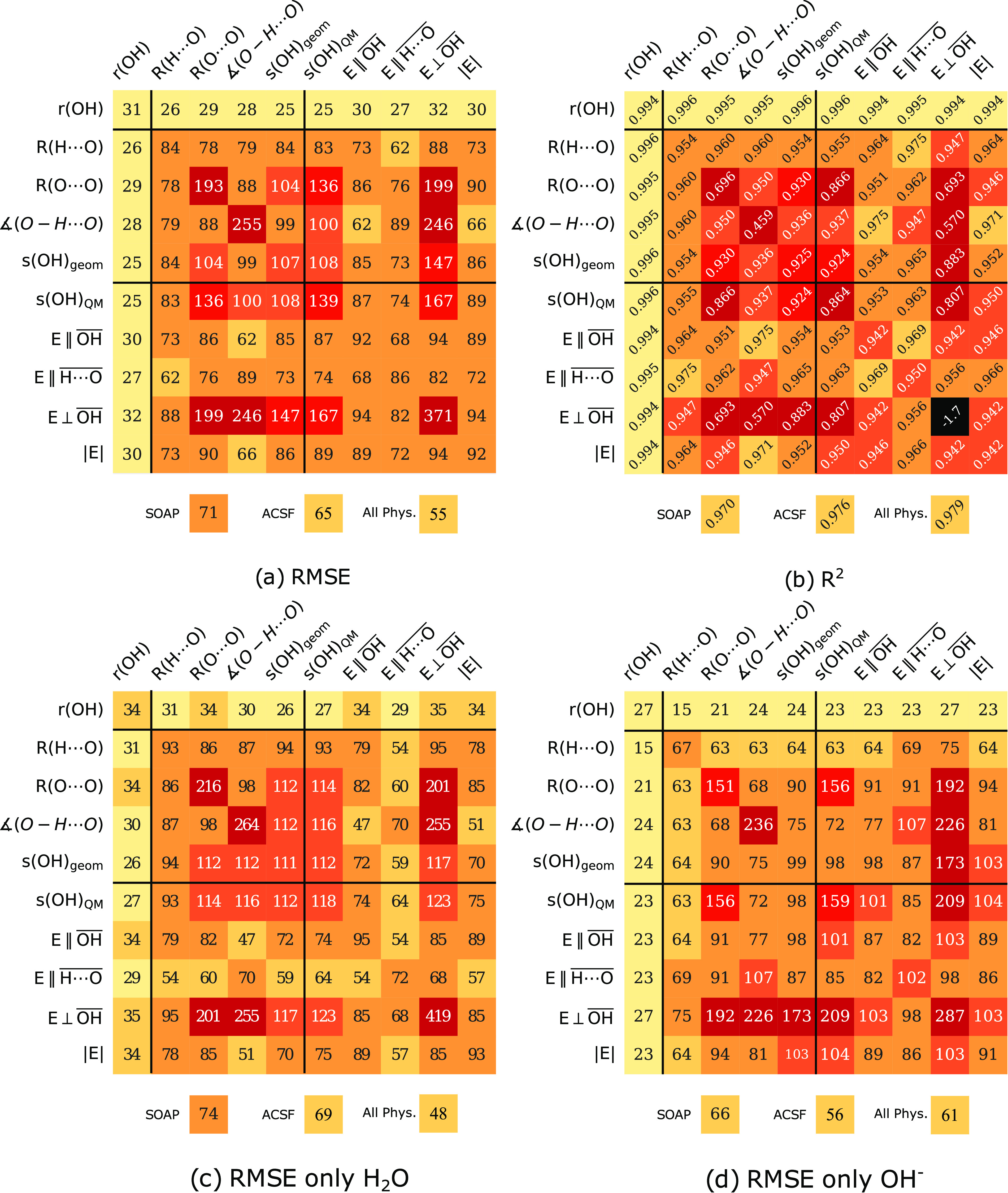
Summary of the performance of the 12 descriptors examined here for surface OH groups: single parameters as well as cross-combinations. The numbers are from Gaussian process regression in all cases.

The RMSE values for the two species H_2_O and OH^–^ separately are given in Figure 7c and d. The two species are seen to perform differently with respect to the two bond order descriptors. For *s*(OH)_geom_, they give rise to rather similar RMSE values (111 and 99 cm^–1^ for H_2_O and OH^–^). *s*(OH)_QM_, on the other hand, yields larger differences between the species (118 and 159 cm^–1^ for H_2_O and OH).

Reference (51), which was also mentioned above, discusses the ν(OH) vs *s*(OH)_geom_ from crystallographic data for bulk crystals. The authors observed a better correlation between ν(OH) and *s*(OH)_geom_ for H_2_O species than for OH^–^, especially for weakly and non-hydrogen-bonded oscillators (i.e., those with large *s*(OH)_geom_ values). They argue that this could either be due to nuclear repulsion shifting the frequencies or the lack of H–Me and H–H bond order terms. In contrast, we do not observe a severe spread in the population of oscillators with large *s*(OH)_geom_ values (∼0.95–1.0). However, we will explore other descriptors that include these contributions in the next sections rather than adding these terms to the bond order expression.

##### 3.2.2.3 Summary

The two chemically intuitive bond order types, estimated either from the atomic positions of the O neighbors (*s*(OH)_geom_) or from the electron density (*s*(OH)_QM_), both perform quite poorly with respect to RMSE for our surface OH groups on metal oxides.

#### 3.2.3 Electric Field Descriptors

##### 3.2.3.1 Context

The idea of representing a chemical environment with an “effective” electric field is commonplace in the scientific literature.^54^ Because (moderately strong) H-bonds are, to a large extent, electrostatic in nature, the “effective” electric field generated by the surrounding environment over the OH oscillator under scrutiny (excluding field contributions from the molecule itself) can be used as a H-bond descriptor. In this context, an early example can be found in the work by Almlöf et al. from 1972^55^ where the OH force constant of water in solid hydrates was determined quantum mechanically using an embedding technique in which an electric field generated from a finite set of point charges was used to mimic the full crystalline environment surrounding the water molecule. Using similar approaches, the correlation between vibrational signals and “effective” electric fields has been explored through vibrational Stark effect spectroscopy.^56^ For OH in particular, the correlation between vibrations and “effective” electric fields has been reported and discussed in the literature in the context of gas-phase species,^57^ bulk liquid water and solvated ions,^58^ and bulk crystalline hydrates and hydroxides,^59^ as well as for surface water in the study by Kebede et al.^24^

##### 3.2.3.2 Results

Here, for each interface system, the atomic charges that generate the electric field were determined from the system’s electron density using the Chargemol/DDEC6 program.^60,61^ Then *for each OH group* in each system (they are all periodic), the electric field at the equilibrium position of its H atom was calculated from DDEC6 charges placed at the positions of all the other atoms (nuclei) “external” to the OH group, using a periodic Ewald summation. The result is an “effective” electric field, which we will refer to as the electric field, denoted *E*. For more details, see the procedure described in ref (24).

First of all, we studied the projection of the external field along the O–H bond for all targeted OH groups. An example best defines a positive electric field direction: when the OH group is surrounded by two charges, + O–H −, then the positive field direction over the molecule is from + to –, that is, from left to right.

The results of our analysis of the capability of *E*∥OH as a descriptor of the OH vibrational frequencies are shown in (Figures 4g and 6g). The RMSE values from the GPR are 95 and 87 cm^–1^ for H_2_O and OH^–^ respectively, and 94 cm^–1^ when the mixed data set is used. Our goodness-of-fit value (*R*^2^) from the GPR treatment is 0.942. This is a little bit less tight than the result we reported above for *R*(H···O), even though the electric field, in a sense, should capture more of the environment. It can be mentioned that Corcelli and Skinner^58^ reported a frequency vs electric field correlation coefficient (*R*) of 0.90 (corresponding to a *R*^2^ value of 0.81) and an RMSE value of 68 cm^–1^ when fitting OH frequencies from cluster fragments from MD snapshots of liquid water to a linear function.

We have already alluded to the fact that the resulting ν(OH) vs *E*∥OH correlation curves differ significantly: the field over OH is always larger for a given frequency, and the two curves are separated by an almost constant offset of 0.03 au.

The lower panel in Figure 6g displays the resulting correlation curves using parabolic fitting functions. The polynomial fittings are practically as good as the GPR results and give RMSE values of 94 cm^–1^ for the H_2_O data, 94 cm^–1^ for the OH^–^ data, and 94 cm^–1^ for the whole data set.

For completeness, the electric field components perpendicular to the internal O–H bond (*E*⊥OH) and the total electric field regardless of direction (|*E*|) were also included. Figures 4h,i and 6h,i report the regression results for these correlations as well, showing that little information is conveyed in the perpendicular direction. In contrast, the total electric field contains almost as much information as the two examples projected along the OH or H···O vectors.

##### 3.2.3.3 Summary

The electric field is the best QM descriptor so far in this paper and predicts the frequency the most accurately when projected along the H···O bond vector.

### 3.3 Regression with Two Descriptors, Heatmaps

In section 3.2.1, we already considered one example of using a combination of two descriptors to perform the regression, namely, the combination of *R*(O···O) distance and the hydrogen bond angle. In this section, we expand on this idea and consider pairwise combinations of *all* physical descriptors used here. The heatmaps in Figure 7 show the *R*^2^ and RMSE values for these pairs of physical descriptors, in all cases from GPR.

All combinations of descriptors that include the internal descriptor *r*(OH) do result in outstanding correlations with low RMSEs, which in all cases (except the uninformative *E*⊥OH) result in slight improvements compared to using just *r*(OH). The effect is most pronounced for OH^–^ where *r*(OH) and *R*(H···O) reduce the RMSE to 15 cm^–1^.

Several combinations of QM and geometric descriptors result in low RMSE, with the lowest being 62 cm^–1^. The low RMSEs can be understood by analyzing the H_2_O and OH^–^ populations separately. Here we find that H_2_O is well described by QM and OH^–^ by geometric descriptors. However, both populations show small RMSE values for combinations of geometric and QM descriptors.

For H_2_O, the best geometric combination gives an RMSE of 86 cm^–1^ while the corresponding value for the QM descriptors is 54 cm^–1^. The combination of geometric and QM descriptors produced RMSE as low as 47 cm^–1^. In the OH^–^ population, the best geometric combination gives an RMSE of 63 cm^–1^, while the corresponding value for the QM descriptors is 82 cm^–1^.

The combination of geometric and QM descriptors produce an overall RMSE of 62 cm^–1^. For the sake of comparison (and completeness), we compare this number to a combination using *all* physical descriptors (excluding *r*(OH) and ∠(O–H···O)^c^), which is 55 cm^–1^ (Figure 7).

In summary, combinations of QM and geometric descriptors lead to the smallest RMSEs.

### 3.4 ML Descriptors: ACSF and SOAP

Making use of the heatmaps in Figure 7 again, we note that the lowest RMSE, for both H_2_O and OH^–^, among the pairwise combinations of geometric descriptors is 78 cm^–1^ (for *R*(H···O) and *R*(O···O)). This number can be compared to 55 cm^–1^ for the overall lowest combination, which includes the quantum mechanically derived electric field. *Can we achieve similar quality with geometrical descriptors, or are full quantum calculations needed to achieve such quality?* To address this question, we performed regression using the high dimensional ML descriptors. We have used the same cutoff as was used in the geometric bond order definition, namely 7 Å.

All the ML results presented in the main text make use of an optimal number of features, defined by the hyperparameters, that minimize the error in the test set (see Supporting Information for more details). Using SOAP, the smallest RMSE is 71 cm^–1^, obtained with *n*_r_ = 1, *n*_l_ = 3. Using ACSF, the smallest RMSE is 65 cm^–1^ with *n* = 7. For more information on the effect of different parameter choices see Figures S6 and S7 in the Supporting Information. However, the error never drops below 65 cm^–1^. This number is very close to the 62 cm^–1^ found for the best quantum mechanically “dependent” pairwise combinations of descriptors in the previous subsection and somewhat higher than the 55 cm^–1^ found for the combination of *all* physical descriptors (excluding *r*(OH) and ∠(O–H···O)). The fact that the RMSE does not surpass the quality of a two-descriptor function using both a geometric and a quantum mechanical descriptor indicates that the quantum environment contains valuable information.

In summary, the RMSE of the ML descriptors show that more information can be obtained by considering the geometric position of all atoms in the external environment than using single descriptors or descriptor pairs. However, in our study, achieving the lowest RMSE is only possible when including the QM descriptors.

### 3.5 The Covalent *r*(OH), a Special Case

The OH equilibrium bond length, *r*_e_(OH), is also a geometric descriptor but is special among the descriptors treated in this paper as it concerns an intramolecular (internal) property of the OH oscillator, and like the vibrational frequency, it is a probe rather than a descriptor of the surroundings. This is because both quantities reflect the intramolecular bond strength of the OH oscillator; a weakening (lengthening) of the equlibrium OH-bond caused by the surroundings is reflected in a decrease of the stretching vibrational frequency.

The plots in Figure 4a and Figure 6a demonstrate that *r*_e_(OH) manages to capture the ν(OH) variation very well, for water as well as for hydroxide. The RMSE value is only 31 cm^–1^ for our compounded test data set from the hydrated-hydroxylated CeO_2_, MgO, and CaO surfaces. Furthermore, the correlation seems to be the same for the bulk and surface data (see Figure 5a). This finding is consistent with the computational work presented in ref (8), which used a similar computational setup but a smaller data set.

We also note that, despite the fact that *r*(OH) achieves an outstanding correlation with the frequency, it is very difficult to infer the vibrational frequency from an experimentally measured structure, even in the case of bulk crystals,^63,64^ due to uncertainties in diffraction-derived *r*(OH) bond lengths. The situation is even worse for surface adsorbed species when surface resolved diffraction techniques, such as low energy electron diffraction,^65^ surface X-ray diffraction,^66^ and atomic beam diffraction^67^ are used. As an illustration to the problem, we can consider the following thought experiment. Consider that we have an uncertainty in the position of H of 0.01 Å (whereas O is perfectly determined); the uncertainty of the descriptors *r*(OH) and *R*(H···O) would both also be 0.01 Å, which can be compared to their typical ranges of values of [0.97, 1.02] and [1.5, 2.5], respectively. The relative errors for these descriptors can be calculated by dividing the uncertainty with the range, and this calculation yields errors of 20% and 1% for *r*(OH) and *R*(H···O), respectively. This simple example highlights that *r*(OH) is particularly sensitive to even small errors and that *R*(H···O) is about a magnitude less so. We expect a similar “insensitivity” as seen for *R*(H···O) in the other descriptors used here. This is further compounded by a numerical experiment that we performed where a perturbation was applied to the position of H for all OH-oscillators presented in this paper; the resulting errors on *R*(H···O), *s*(OH)_geom_, *E*∥H···O, and *r*(OH) are shown in the Supporting Information where the same conclusion can be drawn; that is, that the external descriptors are quite insensitive to uncertainties in the position of H.

Throughout the various populations (H_2_O, OH^–^, H_2_O∪OH^–^), we have found that the internal descriptor *r*(OH), and combinations including *r*(OH), by far outperform external descriptor combinations. *r*(OH) itself gives a RMSE of 31 cm^–1^ whereas the external ones give at best 55 cm^–1^.

Assuming that the assigned external environment can give rise to the whole frequency shift, one would expect external descriptors to yield comparable RMSEs to *r*(OH). Some insights into whether the reason why this is not the case is fundamental or a consequence of a poor choice of external (geometric) descriptor can be obtained with the help of the ACSF descriptor, where instead of excluding the OH oscillator, as was done in section 3.4, we also included the intramolecular distances of the targeted water or hydroxide species. Compared to 65 cm^–1^ when the intramolecular geometry is excluded, the resulting RMSE then becomes 29 cm^–1^, which is very similar to the 31 cm^–1^ for the internal descriptor *r*(OH) alone.

## 4 Conclusions

In this work, we have explored the fully anharmonic vibrational signatures of surface adsorbed water on CeO_2_(111), MgO(001), and CaO(001) using DFT with the optPBE-vdW functional to assess the capability of different classes of structural descriptors regarding their ability to reproduce the vibrational frequency. The test molecule is water (intact and dissociated) on the surfaces of metal oxides. In addition to being hugely important systems, hydrated/hydroxylated metal oxides constitute an interesting class of aqueous systems as they exhibit a plethora of chemical environments for the water and OH species, many of which with challenging geometries, and their OH vibrations span a large frequency range.

Using the protocol of comparing the RMSE of the frequency estimated from a Gaussian process regression of each descriptor versus the observed vibrational frequency, we could compare the descriptors on an equal footing without any *a priori* knowledge of the correlation function. The descriptors were divided into internal and external descriptors with data based on quantum mechanical or geometric quantities. Of the latter, a set of machine learning descriptors were also used.

Among the external descriptors, *R*(H···O) is particularly powerful and can be expressed with a simple analytical function that can be mapped one-to-one, allowing for distance estimations from frequency. Combining geometric and QM descriptors outperformed any strictly geometric or QM descriptor combination by around 25%.

The ML descriptors showed that the single descriptors or descriptor combinations were not detailed enough to cover all the information contained in the geometric positions of the surroundings.

The study of geometric descriptors revealed that H_2_O and OH^–^ bound to metal oxide surfaces are found in a far larger variety of environments than what is observed in bulk crystals, often with highly bent hydrogen bonds. For this reason, the *R*(O···O) descriptor performs significantly worse for surface OH compared to its bulk hydrate and hydroxide counterparts, where hydrogen bonds are close to linear.

By analyzing the performance of the descriptors with respect to the molecular identity of the oscillators, H_2_O and OH^–^, we found that geometric descriptors were, in general, more suited to reproduce the frequency of surface hydroxides. In contrast, the QM-derived descriptors were more suited to reproduce those of water (Figure 7c,d). However, in both sets, the combination of geometric parameters and QM parameters showed an overall reduction, which translated to the joint assessment of RMSE as observed when considering *E*∥H···O + *R*(H···O)) or (*E*∥OH + ∠(O–H···O)).

We envisage that the presented correlation curves and the estimate of their accuracy can be of use in engineering and technology. Moreover, the protocol developed here, where the information content presented for each descriptor is evaluated using the RMSE from a nonparametric Gaussian process, is not limited to the study of surface OH species but could be extended to other descriptors and target properties.

## Supporting Information Available

Influence of parameter choices for Gaussian process regression on the optimization performance (PDF)

## Footnotes

a The difference in cutoff was determined to affect the vibrational frequency in oscillators by less than 10 cm^–1^.

b The remaining panel in Figure 5, panel a, will be discussed in section 3.5.

c *r*(OH) was excluded following the discussion above. However, since *r*(OH) is completely determined from *R*(H···O), *R*(O···O), and ∠(O–H···O), one of these must be omitted from the fit as well to be consistent.
