# Supplementary material for: Predicting Frequency from the External Chemical Environment: OH Vibrations on Hydrated and Hydroxylated Surfaces
Source: J Chem Theory Comput. 2022 Dec 2;18(12):7683–94. doi: 10.1021/acs.jctc.2c00135 (PMC9753585; doi:10.1021/acs.jctc.2c00135)
Supplement: Supplementary file 1 — ct2c00135_si_001.pdf [file ct2c00135_si_001.pdf]

Supporting Information: From H-bond descriptors to SOAP: OH vibrations of hydrated surfaces and their dependence on local structure

Andreas Röckert,* Jolla Kullgren, and Kersti Hermansson*

Department of Chemistry - Ångström Laboratory, Uppsala University, Uppsala, Sweden

E-mail: andreas.rockert@kemi.uu.se; kersti.hermansson@kemi.uu.se

Phone: +46 (0)18-4173766; +46 (0)18 4713767

Sensitivity analysis

Figure 1 show the descriptor sensitivity with respect to a perturbation in the position of H.

Regularization

Figures 2,3,4,5 show how the RMSE from Gaussian process regression are affected by the regularization parameter, α (note that the graph are plotted against $dy = \sqrt{\alpha}$). The kernel and all other specifications are the same as in the main text.

Figs. 2-3 show RMSE for physical descriptors using different dy values.

Fig. 4 show the RMSE for the ACSF descriptor using different n and dy.

Fig. 5 show the RMSE for the SOAP descriptor using different n_r, n_l and dy.

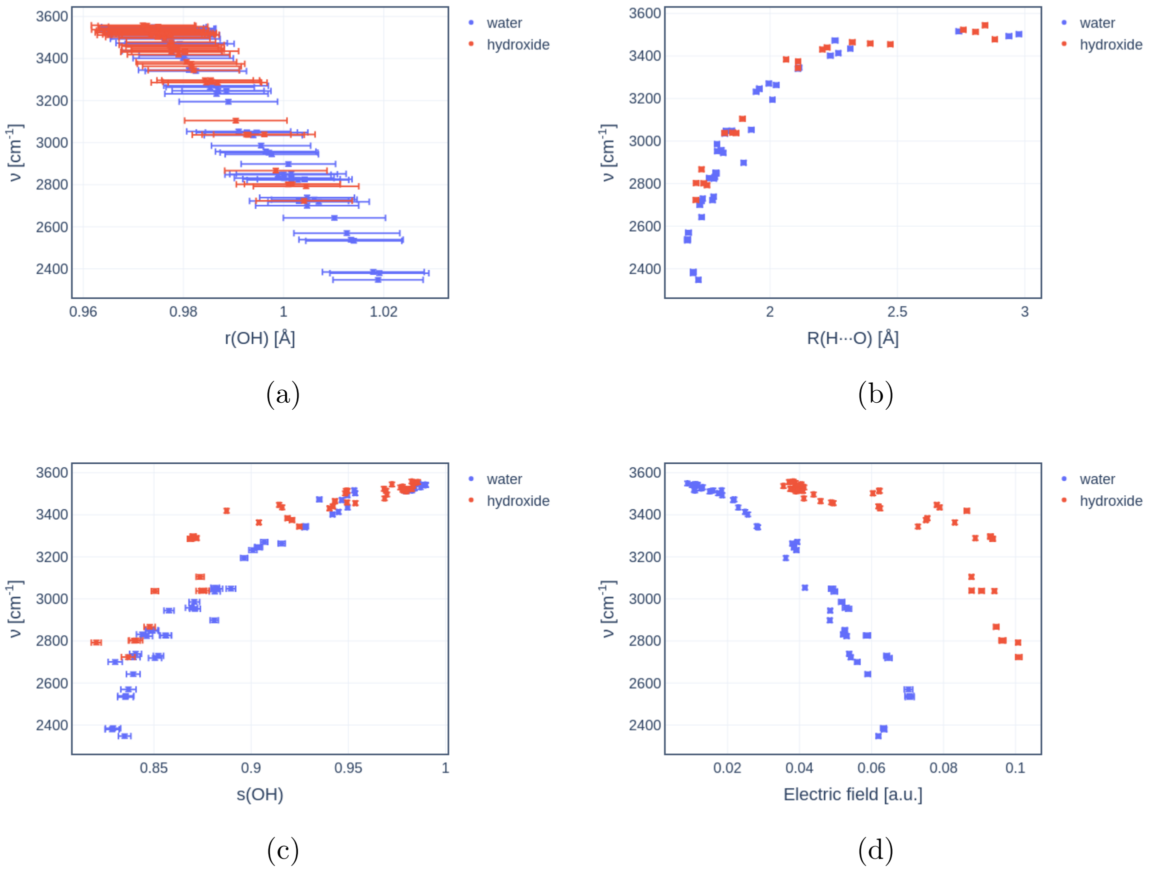

Figure 1: The sensitivity of the descriptors r(OH) (a), R(H $\cdots$ O) (b), s(OH)$_{\text{geom}}$ (c), and E $\parallel \overline{\text{OH}}$ (d) by uncertainties on the position of H. The horizontal error bars reflect the propagated error from perturbing the position of H using a normal distribution with standard deviation $0.01\sqrt{3}$ Å.

Kernels

The kernel used in the main text of this work was selected based on its good balance between flexibility and its tendency towards over-fitting of the data. In this sub-section, we compare the performance of our selected kernel to two other kernels. The kernel selected in the main text is:

$$K(\mathbf{x}_i, \mathbf{x}_j) = C(\sigma^2 + \mathbf{x}_i \cdot \mathbf{x}_j)^4 \tag{1}$$

The two other kernels used in the comparison are, the dot-product kernel without expo-

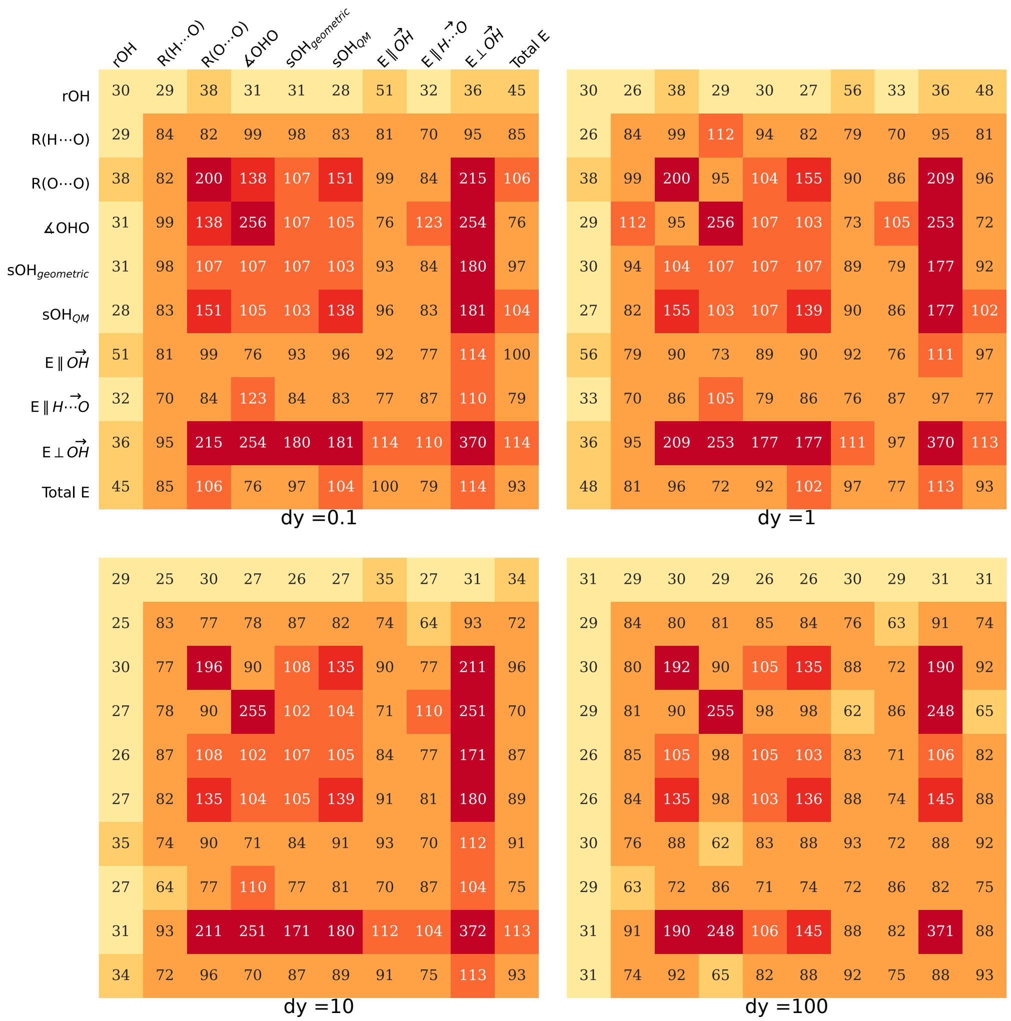

Figure 2: RMSE values for physical descriptors using different dy. Keys in the top left panel applies to the others as well. All units are cm^{-1}.

nentiation:

$$K(\mathbf{x}_i, \mathbf{x}_j) = C(\sigma^2 + \mathbf{x}_i \cdot \mathbf{x}_j) \tag{2}$$

, and the so-called Radial Basis Function (RBF) kernel given by:

$$k(x_i, x_j) = C \exp\left(-\frac{d(x_i, x_j)^2}{2l^2}\right) \tag{3}$$

In the latter, $d(x_i, x_j)$ refer to the euclidean norm and l is a parameter.

Fig. 7 show the RMSE values for GP fitting of $r(HO)$ and ACSF using n=7 with the

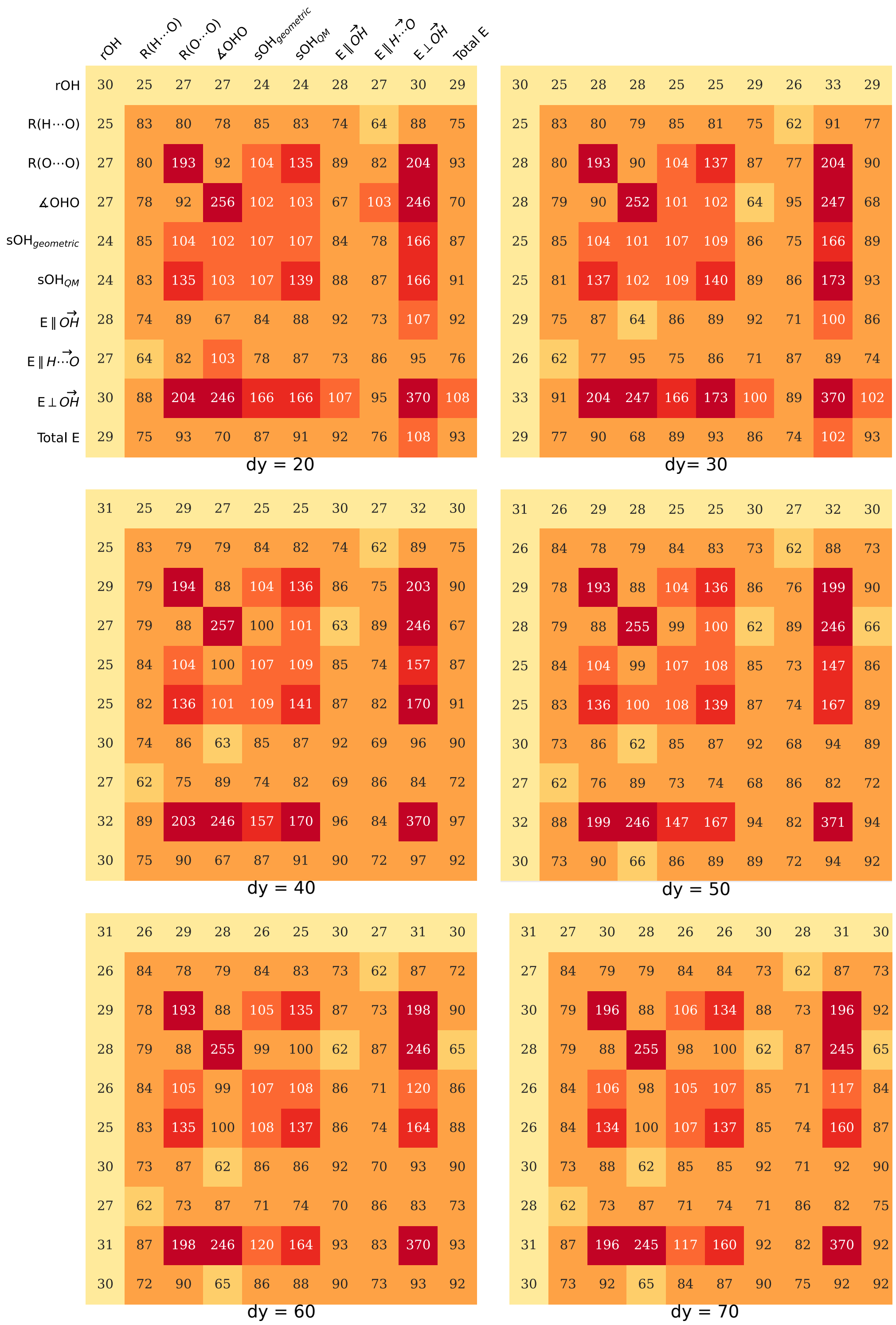

Figure 3: RMSE values for physical descriptors using different dy. Keys in the top left panel applies to the others as well. All units are cm^{-1}.

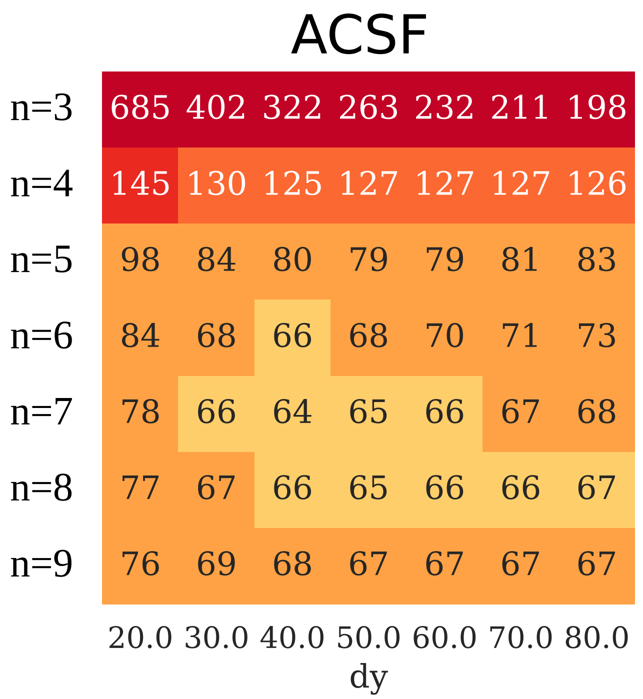

Figure 4: RMSE values for ACSF using different n and dy. All units are cm^{-1}.

different kernels as a function of the Tikhonov regularization α parameter.

Are we over-fitting?

Fig. 6 and 8 show the RMSE for in the training- compared to the test-set using ACSF and SOAP, respectively. The same setting as in main text was used in these plots. A heat-map for RMSE in the training-set of SOAP is shown in Fig. 9.

In order to estimate the potential degree of over-fitting in our procedure we performed an additional test in which 20% of the data was reserved as a ”blind” test-set (blind-set from here on). The remaining 80% of data was then subject to the same treatment as before, albeit this time 20% of the original amount of data was kept for testing. Hence, 60% of the original amount of data was used in the fitting of the model.

Fig. 10 show a comparison of the RMSE in the test-set and the blind-set set using the physical descriptor and pair-wise combinations of them. Clearly, the correlation is strong and indicate that our procedure is robust with respect to over-fitting.

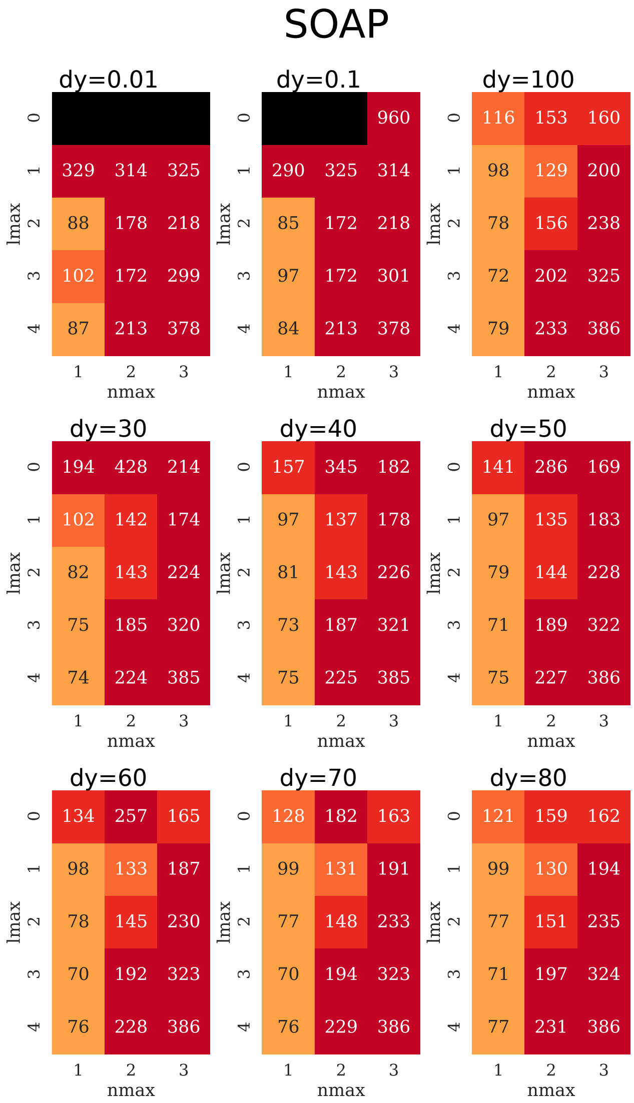

Figure 5: RMSE values for SOAP using different n_r (denoted nmax in the figure), n_l (denoted lmax in the figure), and dy. Black color indicate a RMSE over 1000 cm^{-1}. All units are cm^{-1}.

Fig. 11 show and comparison of the RMSE in the test-set and blind-set as a function of the number of features using the ACSF descriptor. Also in this case, the correlation is seen to be strong.

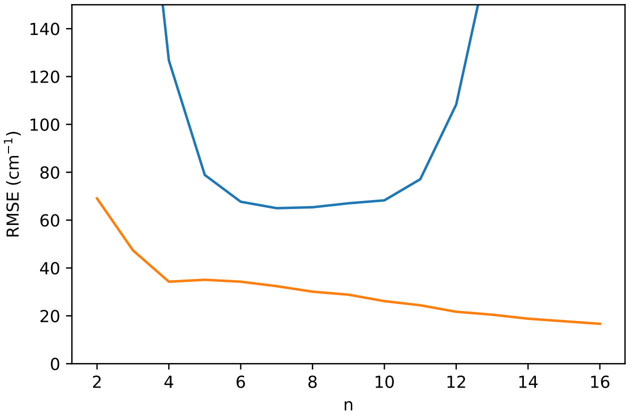

Figure 6: RMSE in the test-set (blue) and training-set (orange) for ACSF using different n. The value of dy in this case was 50 cm^{-1}. All units are cm^{-1}.

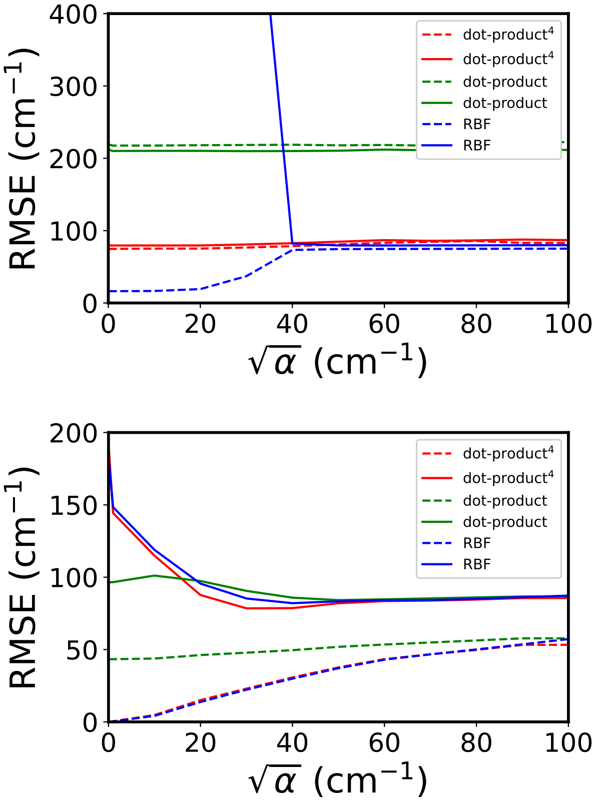

Figure 7: RMSE in the training-set (dashed lines) and test-set (solid lines) for different kernels as a function of $\sqrt{\alpha}$=dy. The descriptor is R(H$\cdots$O) in the upper panel and ACSF using n=7 in bottom panel.

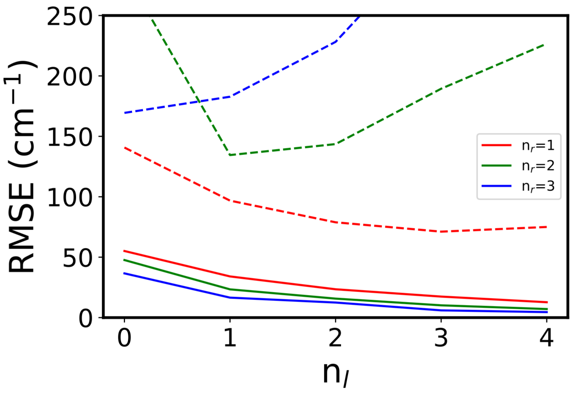

Figure 8: RMSE in the training-set (solid lines) and test-set (dashed lines) for SOAP using different n_r and n_l. The value of dy in this case was 50 cm^{-1}. All units are cm^{-1}.

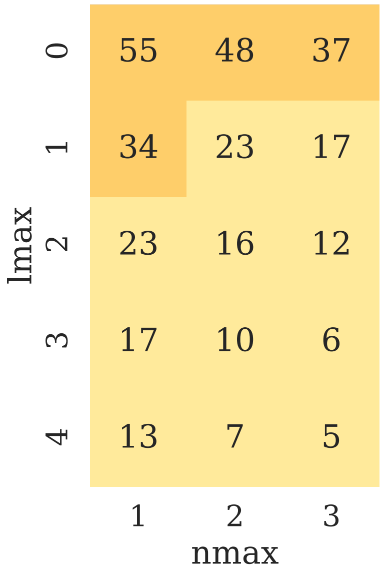

Figure 9: RMSE values in the training-set for SOAP using different n_r (denoted nmax in the figure), n_l (denoted lmax in the figure). The value of dy in this case was 50 cm^{-1}. All units are cm^{-1}.

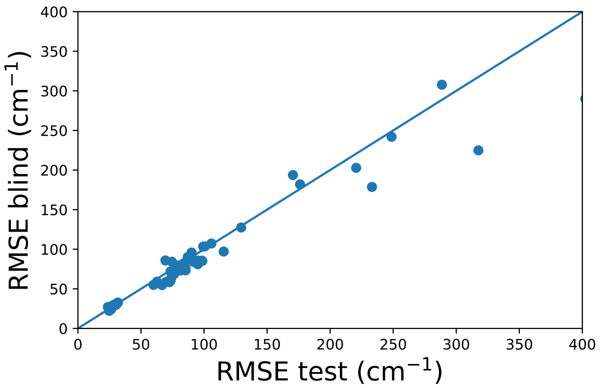

Figure 10: Comparison of the RMSE of a test-set and blind-set on a 60/20/20 split data (train/test/blind). Each point represents a physical descriptor or a combination of physical descriptors.

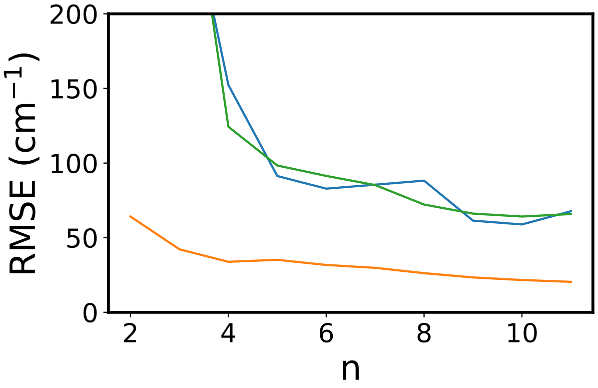

Figure 11: RMSE assessment on a 60/20/20 split data (train/test/blind) as a function of ACSF parameters n. The test-set error (green) and blind-set error (blue) are comparable. In orange is the training-set error.
